# Degenerative suspensory ligament desmitis as a systemic disorder characterized by proteoglycan accumulation

**DOI:** 10.1186/1746-6148-2-12

**Published:** 2006-04-12

**Authors:** Jaroslava Halper, Byoungjae Kim, Ahrar Khan, Jung Hae Yoon, PO Eric Mueller

**Affiliations:** 1 Department of Pathology, College of Veterinary Medicine, The University of Georgia, Athens, GA 30602, USA; 2 Department of Veterinary Pathology, University of Agriculture, Faisalabad, Pakistan; 3 Columbus Children's Research Institute, 700 Children's Drive, Columbus, OH, 43205, USA; 4 Department of Large Animal Medicine, College of Veterinary Medicine, The University of Georgia, Athens, GA 30602, USA

## Abstract

**Background:**

Degenerative suspensory ligament desmitis (DSLD) is a debilitating disorder thought to be limited to suspensory ligaments of Peruvian Pasos, Peruvian Paso crosses, Arabians, American Saddlebreds, American Quarter Horses, Thoroughbreds, and some European breeds. It frequently leads to persistent, incurable lameness and need to euthanize affected horses. The pathogenesis remains unclear, though the disease appears to run in families. Treatment and prevention are empirical and supportive, and not effective in halting the progression of the disease. Presently, the presumptive diagnosis of DSLD is obtained from patient signalment and history, clinical examination, and ultrasonographic examination of clinically affected horses, and is confirmed at post mortem examination. Presently, there are no reliable methods of diagnosing DSLD in asymptomatic horses. The goal of this study was to characterize and define the disorder in terms of tissue involvement at the macroscopic and microscopic levels.

**Results:**

We examined tissues and organs from 28 affected horses (22 Peruvian Pasos, 6 horses of other breeds) and from 8 control horses. Histopathological examination revealed the presence of excessive amounts of proteoglycans in the following tissues removed from DSLD-affected horses: suspensory ligaments, superficial and deep digital flexor tendons, patellar and nuchal ligaments, cardiovascular system, and sclerae. Electron microscopy demonstrated changes in diameters of collagen fibrils in the tendon, and in smooth muscle cells of the media of the aorta compatible with increased cell permeability in DSLD-affected cells. Separation of tendon extracts by gel chromatography revealed the presence of additional proteoglycan(s) in extracts from affected, but not control extracts.

**Conclusion:**

This study demonstrates for the first time that DSLD, a disease process previously thought to be limited to the suspensory ligaments of the distal limbs of affected horses, is in fact a systemic disorder involving tissues and organs with significant connective tissue component. Abnormal accumulation of proteoglycans between collagen and elastic fibers rather than specific collagen fibril abnormalities is the most prominent histological feature of DSLD. Because of this observation and because of the involvement of many other tendons and ligaments beside the suspensory ligament, and of non-ligamentous tissue we, therefore, propose that equine systemic proteoglycan accumulation or ESPA rather than DSLD is a more appropriate name for this condition.

## Background

Injuries to tendons and ligaments are significant causes of lameness and financial losses in the equine industry. They account for nearly a third of all equine injuries that occur during racing, with a reported incidence of 8–43% [1-4]. These tissues heal extremely slowly and the repaired tissue is inferior in elasticity and strength as compared to the original tissue, predisposing to recurrence and repeated injury in up to 80% of affected horses [5-8]. Tendon and ligament injuries may occur as result of acute overloading of the tendon or ligament or secondary to idiopathic degenerative changes [9-11].

Degenerative suspensory ligament desmitis (DSLD) is a heritable, debilitating syndrome recognized in Peruvian Pasos, Peruvian Paso crosses, Arabians, American Saddlebreds, American Quarter Horses, Thoroughbreds, and some European breeds [12]. Affected Peruvian Paso horses demonstrate clinical signs at an earlier age than horses of other breeds [13]. Horses with DSLD typically develop an insidious onset of bilateral or quadrilateral lameness without a history of trauma or performance related injury [13]. Ultrasonography of affected ligaments is characterized by a diffuse loss of echogenicity and an irregular fiber pattern [14-16]. Unique to DSLD, however, is diffuse enlargement of the affected ligaments despite exercise restrictions [12,13]. The pathogenesis of DSLD is incompletely understood. DSLD has been observed to follow familial lines; however, a definitive heritable mechanism has not been established. The presumptive diagnosis of DSLD is obtained from patient signalment and history, clinical examination, and ultrasonographic examination of clinically affected horses, and it is confirmed only at post mortem examination. Presently, there are no reliable methods of diagnosing DSLD in asymptomatic horses.

Though DSLD has been believed to be a disorder confined to the suspensory ligaments (SLs) of the distal limbs of horses, the mechanism of this disease remains largely unknown. The objectives of this study were to 1) identify whether tissues other than SLs are affected by DSLD and 2) characterize the pathology present in such tissues. This study was initiated because pilot findings from our laboratory suggested that the abnormalities in the collagenous tissue of affected horses are not confined to the SLs distal limbs, but may be manifested systemically, in virtually all collagen containing tissues. In this preliminary work abnormal accumulations of yet to be identified proteoglycans appeared to be present not only in the SL, but also in the superficial and deep digital flexor tendons (SDFT and DDFT, respectively), patellar and nuchal ligaments, aorta, coronary arteries and sclerae of DSLD-affected horses.

## Results

### Harvested tissues

Twenty eight horses were referred because of clinical diagnosis of DSLD supported by known bilateral (n = 9) or quadrilateral (n = 17) lameness (the lameness status was not noted in two horses). Lameness was accompanied by an excessively dropped fetlock appearance, and/or palpable enlargement of the branches or body of the suspensory ligament. Ultrasound performed on 19 horses showed evidence of suspensory ligament enlargement with changes in echogenicity, e.g., a hypoechoic, irregular fiber ligament disruption, in at least 14 horses (full ultrasound descriptions were not available in all cases) accompanied by intra-ligamentous calcification in 3 cases. At least 16 horses had known family history of DSLD (Tables 1 and 2). The 28 horses included 22 Peruvian Paso horses (Table 1) and 6 horses of other breeds (2 thoroughbreds, 1 Arab, 1 Hanovarian, 1 Appaloosa and 1 quarter horse, Table 2). In 25 affected horses SDFTs, DDFTs and SLs from all 4 extremities were examined grossly and microscopically at their proximal and midmetacarpal regions; SDFT, DDFT and SL from only one extremity were examined in the remaining three animals. One section from the proximal end and another section from the midmetacarpal end of a tendon or ligament were taken for histological examination. The following tissues were collected from most horses: eyes, portions of nuchal ligament, one of the patellar ligaments, portions of the cardiovascular system, lung, skin, muscle and kidney.

**Table 1:** Peruvian Paso horses affected with DSLD

	**age, sex**	**onset of symptoms**	**clinical**	**family affected**	**legs**	**patella**	**heart**	**PA**	**aorta**	**eyes**	**other tissues**	**ultrasound**
1	1 y, M	6 mos	four	sire	4 mod	ND	cor +	ND	+	both+	nuchal+	2/2 front SLs
2	1 y, F	6 mos	four	sire	4 mild	ND	cor +	ND	+	-	nuchal, lung +	2/2 front SLs
3	18 mo M	6 mos	four	parents	4 mild	ND	ND	ND	ND	ND	nuchal+	ND
4	18 mo M	6 mos	four	parents	4 mod	ND	ND	ND	ND	ND	nuchal+	ND
5	1.5 y, M	at 1 y	four	dam	4 mild	+	-	+	-	both+	lung +	4 SLs
6	2 y, F	birth	four	sire	4 mod	ND	ND	ND	+	ND	ND	¾ SLs
7	3 y, F	6 mos	rear	parents	4 mild	+	cor +	ND	+	both +	nuchal, lung +	4 SLs
8	3 y, M	1 year	front legs	no	4 severe	ND	cor +	ND	+	ND	nuchal+	ND
9	7 y, F	years	four	dam	4 severe	ND	valve+	+	+	ND	lung +	2/4 SLs
10	8 y, M	years	four	sire, foals	1 SL exam, mod*	ND	ND	ND	ND	L eye +	nuchal+	4 SLs
11	9 y, F	at 9 y	rear	dam	4 severe	ND	valve+	+	+	ND	lung +	4 SLs
12	9 y, M	at 6 y	four	no	4 mild	+	cor +	+	+	both+	-	4 SLs
13	9 y, M	years	fallen crest, legs	dam	4 mild	ND	cor +	ND	-	both +	nuchal, lung +	ND
14	11 y, F	years	four	no	1 SL exam, mild*	ND	ND	ND	ND	R eye +	nuchal+	2/2 hind SLs
15	15 y, F	years	four	no	4 mod	ND	ND	ND	+	ND	ND	4 SLs
16	15 y, F	years	front	foal	4 mod	+	cor +	PA+	+	both +	nuchal+	ND
17	15 y, F	years	four	sire, son	4 mod	ND	cor +	ND	+	both +	nuchal, shoulder+	ND
18	18 y, F	years	four	no	1 leg exam, severe*	ND	ND	ND	ND	ND	ND	4 SLs
19	18 y, F	years	rear legs	foals	4 mild	ND	ND	ND	-	R eye +	nuchal, lung +	4 SLs
20	21 y, M	late onset	four	no	4 mod	+	cor +	-	-	R eye +	-	4 SLs
21	21 y, M	years	legs	no	4 severe	ND	cor +	ND	+	R eye +	lung +	4 SLs
22	unknown	unknown	unknown	no	4 mild	ND	-	ND	-	ND	-	ND

Age: age at necropsy; ND: not done; legs: SDFT, DDFT and SL; cor: coronary artery; PA: pulmonary artery; nuchal: nuchal ligament

Mild, mod (moderate), severe: describes the average severity of pathology in examined leg tendons and ligaments; +: accumulation of proteoglycans in other tissues; -: no proteoglycan accumulation

*only limited tissue was available for examination

**Table 2:** DSLD-affected horses of other breeds

	**age, sex, breed**	**onset of symptoms**	**clinical**	**family affected**	**legs**	**patella**	**heart**	**PA**	**aorta**	**eyes**	**other tissues**	**ultrasound**
1	18 mo, F thoro	6 mos	rear legs	no	4 mild	ND	ND	ND	+	ND	nuchal+	ND
2	5 y, M Han	16 mo	forelimbs	no	4 severe	ND	Cor +	ND	+	R eye +	nuchal+	front SLs
3	5 y, F, Appal	6 mos	four	sire	4 severe	yes	cor N	ND	+	both+	nuchal, lung +	2/2 hind SLs
4	8 y, M thoro	6 yr	rear legs	sire sudden death	4 mild	ND	ND	ND	+	-	nuchal+	rear legs
5	16 y, M, Arab	unknown	years	no	4 mild	ND	valve+	ND	+	-	-	ND
6	18 y, F quarter,	unknown	rear legs, lung sounds	no	4 mild	ND	cor	ND	+	both+	nuchal, lung +	rear legs

Age: age at necropsy; ND: not done; legs: SDFT, DDFT and SL; cor: coronary artery; PA: pulmonary artery; nuchal: nuchal ligament; Han: Hanovarian; Appal: Appaloosa; thoro: thoroughbred

Mild, mod (moderate), severe: describes the average severity of pathology in examined leg tendons and ligaments; +: accumulation of proteoglycans in other tissues; -: no proteoglycan accumulation

In addition, tissues from 8 control horses of both sexes, different breeds (3 quarter horses, 1 Percheron, 1 Arab, 1 Tennessee walking, 1 pony and 1 Peruvian Paso) and ages and with no known medical problems (Table 3) were examined as well.

**Table 3:** Control horses

	**age, sex, breed**	**onset of symptoms**	**clinical**	**family affected**	**legs**	**patella**	**heart**	**PA**	**aorta**	**eyes**	**other tissues**	**ultrasound**
1	young, F, quarter	NA	healthy	no	2 fibr. tendons, mild	ND	-	ND	-	ND	ND	ND
2	5 mo, M, Percheron	NA	healthy	no	foci in 4 legs	ND	-	ND	-	-	nuchal+	ND
3	6 y, F, quarter	NA	Cushing	no	fibrosis in four, mild	-	-	ND	-	-	lung +	ND
4	8 y, F, quarter	NA	healthy	no	foci in 4 legs	-	-	ND	-	-	-	ND
5	8 y, M, Tennessee walking	NA	slight fever	no	fibrosis in four, mild	-	-	ND	-	-	nuchal+	ND
6	9 y, F, Arab	NA	healthy	no	foci in 4 legs	-	-	ND	-	-	nuchal+	ND
7	9 y, F, Per Paso	NA	healthy	no	four, mild	+	cor +	ND	-	-	nuchal, lung +	ND
8	10 y, M, pony	NA	healthy	no	foci in 4 legs	-	cor +	ND	-	-	nuchal, lung +	ND

Age: age at necropsy; ND: not done; legs: SDFT, DDFT and SL; cor: coronary artery; PA: pulmonary artery; nuchal: nuchal ligament

Mild, mod (moderate), severe: describes the average severity of pathology in examined leg tendons and ligaments; +: accumulation of proteoglycans in other tissues; -: no proteoglycan accumulation

### Pathology of tendons and ligaments

**Figure 1:**
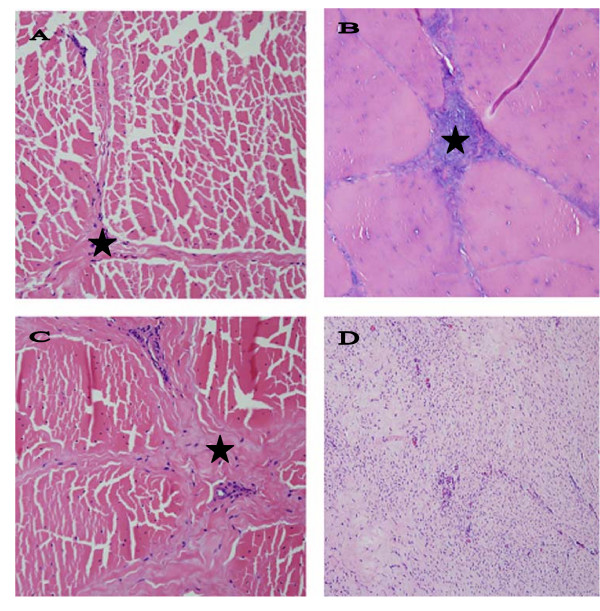
**Comparison of normal and DSLD-affected tendons**. A. Only thin septa (*) separate bundles of collagen and elastic fibers in a normal tendon. Hematoxylin & eosin, magnification × 200. B. A section of DSLD-affected tendon reveals PG deposits (*) between collagen fibers and in septa. Hematoxylin & eosin, magnification × 200. C. A section of tendon from a horse without DSLD shows the presence of fibrosis or scar tissue (*) between collagen fibers and in septa. Hematoxylin & eosin, magnification × 200. D. A proliferative lesion found in one DSLD case consists of swirls of active fibroblasts in young, well vascularized tissue. Hematoxylin & eosin, magnification × 200.

Normal tendons and ligaments have a uniform, grayish pink color and are pliable on palpation. In normal tendons and ligaments fascicles and bundles of collagen fibers are separated by thin endotenon septa that contain only a few small blood vessels in SDFTs and DDFTs, and in patellar and nuchal ligaments (Figure 1A). In addition, small islands of striatus muscle are interspersed with bundles of collagen fibers of the suspensory ligaments; a reminder of the evolutionary past when SL was more a muscle rather than a ligament (or tendon strictly speaking).

**Figure 2:**
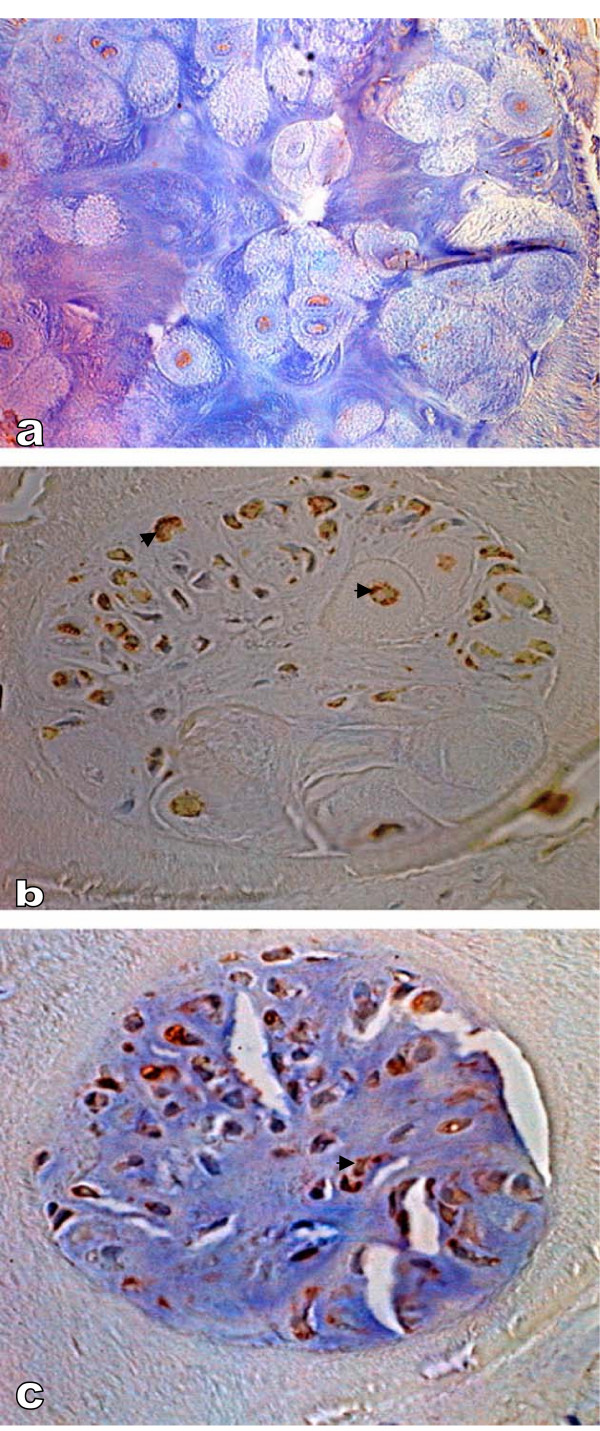
**Immunostaining of metaplastic cartilage**. A. Immunostaining reveals very little decorin, magnification × 500. B. The cartilage reveals the presence of aggrecan (▶) in the cytoplasm, magnification × 400. C. The same chondrocytes were also positive for biglycan (▶), magnification × 400. Countestain: hematoxylin.

All SDFTs, DDFTs and SLs were found to be affected in all four extremities in 25 DSLD horses and the one extremity that was examined in the 3 remaining DSLD horses (no. 10, 14 and 18, Table 1). Usually but not always both proximal and midmetacarpal portions of the tendons and ligaments were affected. Grossly the affected tendons and suspensory ligaments felt firm and inflexible, and contained foci of white tissue. The histopathological findings consisted of deposits of acellular amorphous material staining blue with hematoxylin & eosin stain between collagen fibers, in septa and around blood vessels (Figure 1B). In some cases the septa were also infiltrated with small blood vessels. In many tendons and ligaments the diffusely distributed proteoglycans gave the collagen a diffuse blue tinge. The extracellular matrix of affected septa and blood vessels stained for decorin, biglycan and aggrecan. Small foci of cartilage or calcifications were occasionally dispersed among collagen fibers. The cytoplasm of chondrocytes forming these foci stained strongly for biglycan and aggrecan, considerably less for decorin (Figure 2). No immunostaining was noted in the extracellular matrix of the cartilage.

In addition, rear suspensory ligaments in one DSLD horse (case no. 6, Table 2) revealed the presence of exuberant cellular areas consisting of fibroblasts in young connective tissue, with very little collagen present but with incipient PG accumulation (Figure 1D). Hypercellular septa due to proliferation of fibroblasts and increased number of blood vessels together with mild deposits of PGs were observed in a young Peruvian Paso (1.5 yr old male, case no. 5, Table 1). In one horse (no. 5, Table 2) actively proliferating synovium containing numerous blood vessels was observed in the midmetacarpal portion of the left rear suspensory ligament (data not shown). By light microscopy there was complete absence of inflammatory cells in all types of lesions, including the exuberant proliferating foci.

The histopathological changes in leg tendons and SLs were divided into mild, moderate and severe as indicated in Tables 1, 2. Because so many tendons were evaluated the grading represents an overall degree of severity of disease for each animal. It was based on the worst lesions present in several tendons and SLs in extremities from the same animal. Nine cases were quantified as mild (based on up to 10% of tissue affected by lesions), 8 as moderate (i.e., up to 30% of tissue affected by lesions) and 5 as severe (i.e., more than 30% of tissue affected by lesions). It is interesting that there was little correlation between the severity of the lesions and signalment or clinical history, such as pain or lameness (data not shown).

**Figure 3:**
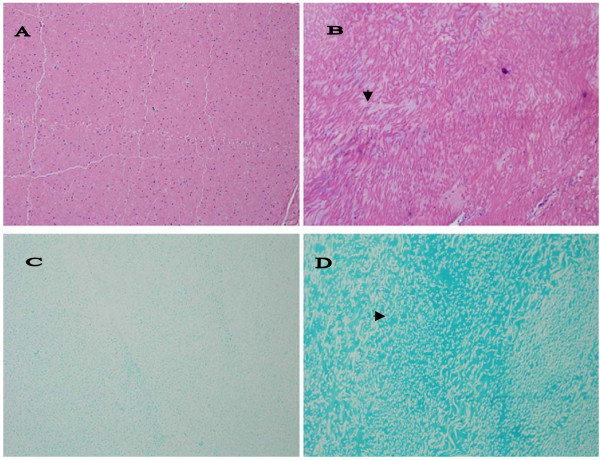
**Histopathological changes in nuchal ligament**. A. Only thin septa separate bundles of collagen and elastic fibers in a normal nuchal ligament. Hematoxylin & eosin, magnification × 200 (× 200). B. In DSLD – affected tissue streaks of proteoglycans (▶) separate bundles of collagen and elastic fibers. Hematoxylin & eosin, magnification × 200. C. Alcian blue stains very lightly normal nuchal ligament, magnification × 200. D. PGs accumulated among bundles stain intensively with alcian blue (▶) in DSLD-affected nuchal ligament.

Similar histopathological changes, i.e., either focal deposits of PGs or areas of diffuse blue tinge, were present in all examined patellar (6 horses) and majority of nuchal ligaments (17/19 examined samples, Figure 3B) from DSLD horses. Figure 3D demonstrates that this material gave a strong reaction with alcian blue, indicating that it consists of proteoglycans (PGs).

Interestingly, histological signs of old tendon injuries were found in all examined control horses in at least one tendon (or suspensory ligament), and in all four extremities in 5 horses (Table 3). Unlike the DSLD tendons and suspensory ligaments, these tendons and SLs containing lesions, however, were pliable on palpation and no gross lesions were apparent. Histologically, the injury was characterized by the presence of fibrosis accompanied by focal accumulation of proteoglycan material most commonly in the midmetacarpal portion of the tendon or suspensory ligament (Figure 1C). The lesions were less extensive than the lesions in the DSLD tendons and were either mild or just consisted of small foci (Table 3). One horse, a 9 year old Peruvian Paso, exhibited histopathological changes in tendons of all 4 extremities, the nuchal ligament and other tissues consistent with DSLD (horse no. 7, Table 3).

Nuchal ligaments from four control horses revealed the presence of small foci of PGs. In general, the foci were much smaller than the PG deposits found in DSLD ligaments and were limited to only a small area of the nuchal ligament. Another control horse (no. 7, Table 3) showed more extensive changes in the nuchal ligament and other tissues consistent with diagnosis of DSLD (see above).

### Electron microscopic changes in DSLD-affected tendons

**Figure 4:**
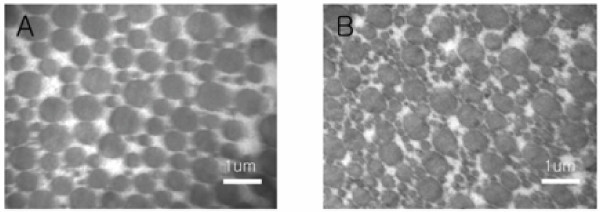
**Electron micrographs of normal and DSLD-affected tendon**. A. A cross-section of normal tendon reveals that most collagen fibrils have fairly large diameters. B. A marked increase in small collagen fibrils was observed in cross-sections of DSLD-affected tendon.

Cross-sections from midmetacarpal portions of SDFTs were examined. The majority of collagen fibrils in normal tendon had a bimodal distribution, most fibrils having a fairly large diameter. Only a few fibrils with small diameter were observed (Figure 4A). Sections from DSLD-affected tendon showed a marked increase in the number of mostly small fibrils dispersed among larger fibrils (Figure 4B).

### Sepharose CL-2B chromatography of tendon proteoglycans

**Figure 5:**
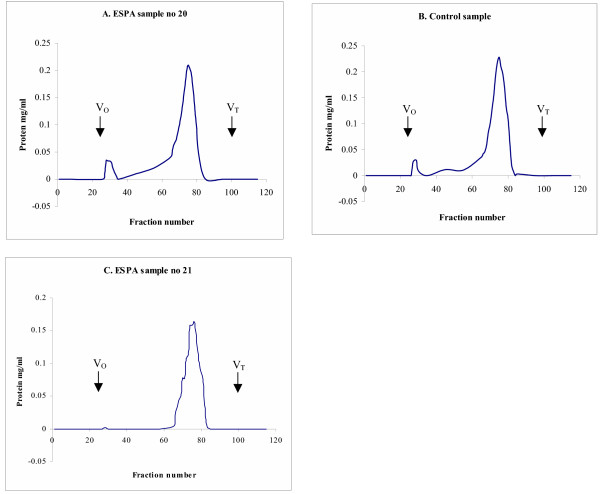
**Sepharose CL-2B chromatography of PGs in extracts from normal and DSLD-affected tendons**. Guanidium HCl extracts from midmetacarpal portions of SDFTs from 2 affected horses (no. 20 and 21, Table 1) and from one control horse (no. 1, Table 3) were separated on a molecular sieve Sepharose CL-2B column (1.3 × 110 cm, equilibrated and eluted in 4 M guanidine HCl) at 0.1 ml/min. The majority of PGs eluted in one wide peak (fractions 66–81).

**Figure 6:**
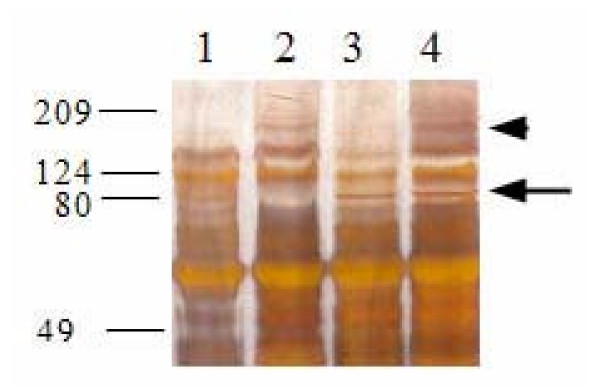
**SDS-PAGE of Sepharose CL-2B peaks**. Aliquots of pooled Sepharose CL-2B fractions were precipitated with 100% ethanol, dissolved in 15 μl of 100 mM Tris buffer, adjusted to pH 8.0 with concentrated acetic acid and digested with 20 mU of chondroitinase ABC at 37°C for 24 h. Chondroitinase ABC digested samples and non-digested replicate aliquots were separated on silver stained 10% SDS-polyacrylamide gels. Lane 1: untreated sample from control SDFT no. 1; lane 2: untreated sample from DSLD SDFT no.8 (Table 1); lane 3: chondroitinase ABC treated sample from control SDFT no. 1; lane 4: chondroitinase ABC treated sample from SDFT no. 8 (Table 1).

We performed an initial analysis of proteoglycan composition in the midmetacarpal portion of the SDFT. Because this analysis is time and labor intensive, it was done only on a limited number of samples. All Sepharose CL-2B chromatographs from one control (no. 1, Table 3) and two affected horses (no. 4 and 8, Table 1) showed separation into one main peak (Figure 5). Sodium dodecyl sulfate polyacrylamide gel electrophoresis (SDS-PAGE) on fractions pooled from the main peak from the extract from DSLD tendons showed a distinct band at ~180 kDa (Figure 6, arrowhead). The presence of the band seen only in abnormal tendons was independent of treatment with chondroitinase ABC. Treatment with chondroitinase ABC led to the appearance of an 80 kDa band in the DSLD sample (Figure 6, arrow). Interestingly, the control sample revealed a band in the same location. Again the presence of the band was independent of treatment with chondroitinase ABC.

### Changes in other tissues and organs

Focal deposits of proteoglycans were found in several other collagen containing tissues in DSLD affected horses, such as the aorta, pulmonary and coronary arteries and sclera. No tissues besides tendons and ligaments (see above) contained exuberant cellular connective tissue.

**Figure 7:**
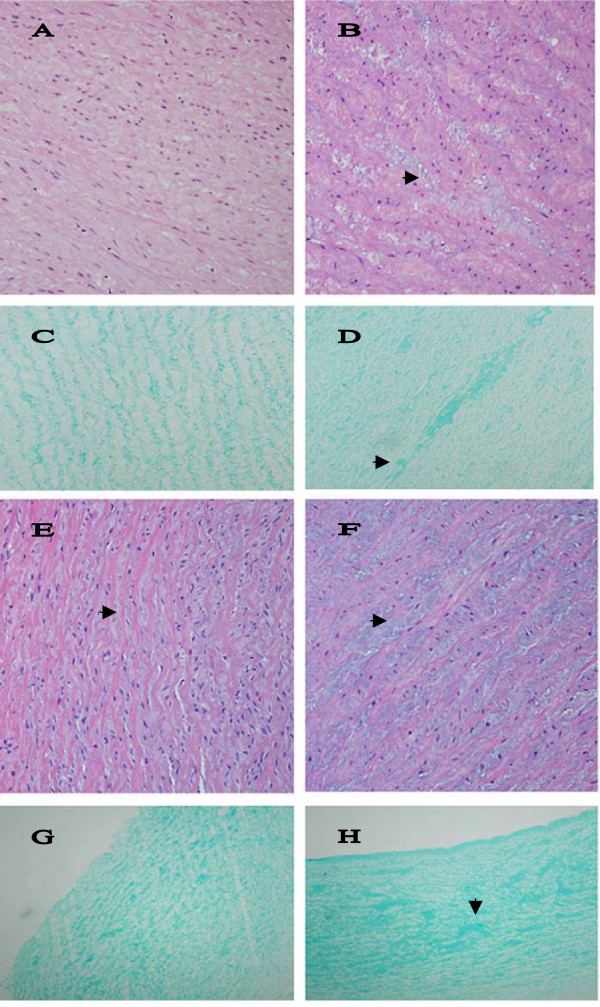
**Histopathological changes in arteries**. A. The media of normal aortic arch shows orderly aligned elastic fibers and very little PGs between them. Hematoxylin & eosin, magnification × 200. B. Small pools of PGs (▶) separate fibers and cells in the media of DSLD-affected aortic arch. Hematoxylin & eosin, magnification × 200. C. Alcian blue stains material aligned closely with elastic fibers in normal arch, magnification × 200. D. Pools of PGs (▶) stain strongly with alcian blue in the media of DSLD-affected arch, magnification × 200. E. The media of normal coronary artery shows orderly aligned elastic fibers separated by thin layers of PGs (▶) between them. Hematoxylin & eosin, magnification × 200 (× 200). F. Small pools of PGs (▶) separate fibers and cells in the media of DSLD-affected coronary artery. Hematoxylin & eosin, magnification × 200. G. Alcian blue stains material aligned closely with elastic fibers in the media of normal coronary artery, magnification × 100. H. Pools of PGs (▶) stain strongly with alcian blue in the media of DSLD-affected coronary artery, magnification × 100.

**Figure 8:**
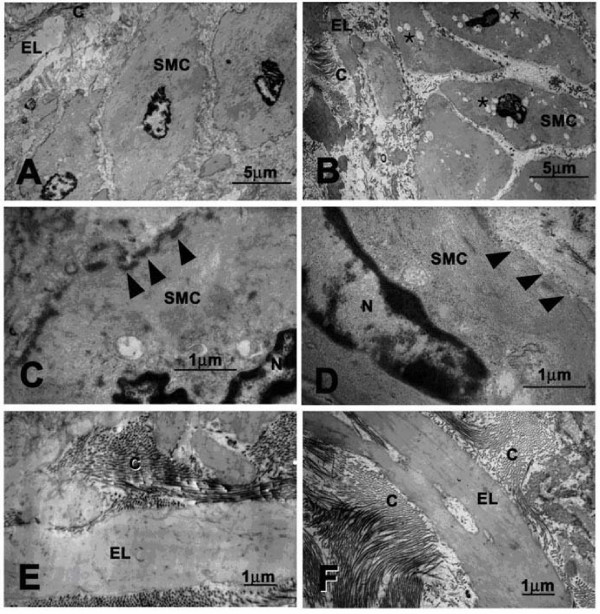
**Ultrastructural features of normal and DSLD-affected aorta**. A. Normal smooth muscle cell from media from a healthy aorta. B. A smooth muscle cell from the media of DSLD-affected aorta reveals the presence of numerous cytoplasmic vacuoles (*). C. normal smooth muscle cell has a well defined cell membrane (▶). D. The cell membrane of a diseased media is disrupted and missing in places (▶). E and F. Organization of collagen fibrils and elastic fibers is similar in normal (E) and DSLD aorta (F). SMC: smooth muscle cell, EL: elastic lamina, C: collagen fibrils.

The walls of affected blood vessels showed irregular thickening of the intima and media due to the presence of proteoglycans (Figure 7B and 7F). This was accompanied by collagen and elastic fiber bundle disorganization that was due to separation of fibers by small pools of proteoglycans staining strongly with alcian blue (Figure 7D and 7H). Three examined heart valves from DSLD-affected horses were vascularized. One examined control heart valve was normal (data not shown). Transmission electron microscopy revealed the presence of numerous small vacuoles in the cytoplasm of smooth muscle cells in media from DSLD aorta (Figure 8B). The cell membrane of these cells was thinner and discontinuous (Figure 8D). However, the collagen fibrils and elastic fibers exhibited similar features and appeared to be intact in normal and DSLD aortas Figure 8E and 8F).

Unilateral or bilateral scleral involvement was observed in 12/18 of DSLD horses where eyes were examined. The changes consisted of blue discoloration of collagen fibers (data not shown).

In 10 DSLD horses (out of 19 examined) varying in age from 1 to 21 years, and in 3 out of the 7 examined control horses minor lung lesions were present consisting of mild peribronchial, perivascular and septal fibrosis (data not shown). A small collection of mostly chronic inflammatory cells were observed in the peribronchial fibrous tissue of the older, but not of the younger horses (1, 1.5 and 3 years of age). In addition, the fibrosis was more prominent in these 3 young horses, particularly around small arteries. In one young horse (no. 2) small deposits of PGs were discernible in alveolar walls.

Kidney, skeletal muscle and skin appeared normal in all horses. Only one DSLD horse (no. 5, Table 2) was diagnosed as having chronic idiopathic granulomatous dermatitis, panniculitis and rhabdomyositis in addition to DSLD.

None of the control horses revealed systemic involvement except for a 9 year old Peruvian Pasofino mare (no.7, Table 3). The pathology in this horse was consistent with DSLD (see above).

## Discussion

Historically, the pathology associated with the clinical syndrome DSLD has been thought to be limited to the suspensory ligaments of the distal limb of horses. The findings of this study suggest that DSLD is in fact a systemic disorder involving many tissues and organs with a significant connective tissue component. Tissues with histological lesions in addition to the suspensory ligament documented in this study include deep and superficial digital flexor tendons, patellar ligaments, aorta, coronary arteries, nuchal ligaments, and ocular sclerae. In light of these observations, a more appropriate term for this disease process may be equine systemic proteoglycan accumulation (ESPA).

While specific collagen fibril abnormalities have been reported to be a consistent feature of DSLD [13], our observations suggest that abnormal accumulations of proteoglycan between collagen fibers, within the tendon matrix, and between elastic fibers in the blood vessels are the most consistent and prominent histological feature associated with DSLD affected horses rather than primary collagen fibril abnormalities.

The staining properties of material accumulated in connective tissues, the presence of cartilage metaplasia and the appearance of an 80 kDa band after chondroitinase ABC treatment in gel chromatography-separated tendon extracts indicate that DSLD is indeed due to deposits of a yet to be identified PG(s). The difference in immunostaining for PGs between septa and blood vessels on one hand and newly formed cartilage on the other (i.e., extracellular vs cytoplasmic pattern of staining) can be attributed to the fact that the cartilage foci were small and cellular, with only relatively small amounts of extracellular matrix. In other words the cartilage foci were young and thus in the process of formation of extracellular matrix. It is likely that the extracellular presence of PGs in the septa within the tendon indicates long standing lesions. Because of technical difficulties we did not attempt to "uncover antigens" during the immunostaining procedure (that process leads to the tendon sections slipping off the slide), and it is possible that the immunostaining was incomplete.

We interpret the appearance of the 80 kDa band after chondroitinase ABC treatment a result of digestion of GAGs attached to a PG(s) of higher M_r _(likely around 100 kDa). It is likely that the carbohydrate component is chondroitin or dermatan sulfate [17]. Further studies are in progress to identify this and the 180 kDa band which appears only in the DSLD samples. The marked increase in small collagen fibrils in DSLD tendons is most likely secondary to qualitative changes in the synthesis of PG(s). PGs are involved in regulation of collagen fibrillogenesis, including the rate of formation and final sizes of fibrils [18]. For example, decorin, the most common PG in the tendon, limits collagen fibril growth, and, thereby, directs tendon remodeling in response to tensile forces. It has been shown that knock-out mice deficient in decorin have fragile connective tissues [19]. Similarly, the absence of biglycan and/or fibromodulin, two proteoglycans related to decorin, in knock-out mice prevented formation of mature collagen fibrils and led to ossification of tendon fibrocartilage [20]. Whether the accumulated proteoglycan(s) in DSLD is identical to any of these PGs remains to be determined.

The proliferative tendon lesions found in three horses with DSLD are significant as they represent in all likelihood early lesions which eventually progress to a less cellular stage characterized by increasing PG accumulation. Characteristically, no inflammatory or fibrotic changes accompanied these deposits or proliferative lesions. We hypothesize that the proliferating fibroblasts secrete PGs which then accumulate in tissues. The stimulus for the proliferation of fibroblasts and the subsequent production of proteoglycans is unknown. The proliferation of fibroblasts and growth of the exuberant connective tissue may explain the presence of pain in the early stage of the disease.

Though flexor tendon microinjuries and subsequent histological lesions can be found in virtually all horses, the tendon lesions in horses afflicted with DSLD differed significantly from "control" horses. All 28 horses with DSLD had histopathological lesions in all SDFTs, DDFTs and SLs. It is of interest that in our study many control SDFTs and DDFTs had signs of old microinjuries consisting mostly of small foci of fibrosis, i.e., regular scar tissue and small deposits of PGs in the midmetacarpal (and proximal) region. This is different from other studies claiming the sparsity of injuries to DDFT in normal horses [21,22]. The lack of systemic involvement in control horses is also indicative that they did not have DSLD.

It is not clear whether the changes observed in the blood vessels and eyes progress over time and lead to clinical manifestations. Several horse breeders brought to our attention that they have encountered horses with a record of clinically diagnosed DSLD dying suddenly without a precipitating disease. Abnormalites in connective tissues components of the aorta or major vessels may predispose to rupture of aortic aneurysm and sudden death [23,24]. Further epidemiologic studies are necessary to determine the clinical significance of these findings.

DSLD is thought to run in families and bears some similarity to several hereditary diseases afflicting connective and musculoskeletal tissues in people. The first is called Marfan's syndrome, which affects joints, the aorta, heart valves and eyes. Affected individuals are typically tall, have heart and eye problems and frequently die of a catastrophic rupture of the aorta. The genetic and biochemical defect of Marfan's syndrome is a mutation in the gene for fibrillin-1, an extracellular matrix protein involved in proper organization of fibrils [23]. Because fibrillin-1 regulated activation of transforming growth factor β is impaired in Marfan's patients and fibrillin deficient mice, it is quite likely that accumulation of proteoglycans causing the prolapse of mitral valve, a prominent feature in Marfan's syndrome, is one of the consequences [24]. Whether secondary changes in regulation of growth factors and other mediators play a role in DSLD is not known at the present time. Elastic fragmentation and cystic changes can be found in Marfan's syndrome and Erdheims' cystic medionecrosis [25] (and also in DSLD). In addition the EM findings of small vacuoles in the cytoplasm of smooth muscle cells in the media from DSLD aorta and of thinner and discontinuous membrane of these cells appeared to be similar to ultrastructural changes observed in endothelial cells in aortas from patients with Marfan's syndrome and Erdheim's disease and are indicative of increased cell permeability [26].

The second human disorder or group of related disorders is Ehlers-Danlos syndrome. People with this disorder experience increased fragility of the skin and hyperflexibility of the joints. The skin may be very loose, forming folds, and prone to scarring. Because Ehlers-Danlos syndrome comprises a group of clinically similar diseases rather than one defined entity, its pathogenesis is more complicated. It is likely that different mutations lead to different forms of Ehlers-Danlos syndrome [27]. Mutation in the gene encoding type III collagen leads to the most severe form of Ehlers-Danlos syndrome, type IV [28]. Patients with this form often develop and die of rupture of large blood vessels, uterus or intestines [29,30].

The third, called Williams syndrome is characterized by distinctive facial features, mental retardation, and cardiovascular disease. Sudden death often in young people is associated with stenoses and other abnormalities of coronary arteries [31]. Other cardiovascular malformations include aortic stenosis and arch hypoplasia [32]. The underlying defect is a mutation in the gene encoding for elastin (*ELN*) [33]. The fourth entity, Caffey syndrome or infantile cortical hyperostosis is characterized by subperiosteal bone formation, some Ehlers-Danlos features (e.g., joint hyperlaxity and hyperextensible skin), but also by a localized inflammatory response. The symptoms are attributed to a mutation in the gene encoding the α1(I) chain of type I collagen (*COL1A1*) [34]. It is interesting to note that some DSLD horses or their offspring develop osteopetrosis (David Burrell, personal communication).

As far as we know none of these human hereditary disorders is known to be caused by a defect in proteoglycan biochemistry or genetics, thus making it different from DSLD. However, because all mutations occur in genes encoding for structural components of tendons and blood vessel walls (e.g., collagen, elastin, fibrillin) that contribute to biomechanical integrity of these tissues, the diseases share certain similarities in their presentation.

Current experiments in our laboratory are directed at the characterization and identification of the specific proteoglycans involved in DSLD. Further characterization and understanding of the pathogenesis of DSLD will allow us develop diagnostic tests to identify asymptomatic horses and help prevent the propagation of the syndrome through a selective breeding program.

## Conclusion

The findings of this study demonstrate that so called degenerative suspensory ligament desmitis (DSLD) thought to be limited to suspensory apparatus is actually a systemic disorder affecting tissues with a high content of connective tissue, such as tendons and other ligaments, blood vessels and sclerae. Initial investigations suggest that accumulation of still to be identified proteoglycans between collagen and elastic fibers and around small blood vessels is the underlying pathology. Due to the systemic nature of the disease we feel that the term equine systemic proteoglycan accumulation or ESPA is more appropriate than DSLD.

## Methods

### Cases and tissue collection

All horses were donated for research to the College of Veterinary Medicine, The University of Georgia, Athens, GA. All procedures were approved by the Animal Care and Use Committee, University of Georgia, IACUC# A2001-10120). Twenty-eight horses clinically diagnosed with DSLD (22 Peruvian Paso horses and 6 horses of other breeds) were compared to 8 horses donated for reasons other than lameness (control group) using light and electron microscopy, and gel chromatography. Postmortem examination was conducted immediately following euthanasia and tissues of interest were either processed for histopathological and electron microscopy studies, or frozen at -20°C until used for biochemical analysis. Superficial and deep digital flexor tendons and suspensory ligaments were removed proximally at the level of the proximal third metacarpus (= proximal portion) to the level of the metacarpophalangeal joint above the fetlock (or above the bifurcation of the SL, labeled by us as the midmetacarpal portion). One section from the proximal end and another section from the midmetacarpal end were taken for histological examination. In many horses the following tissues were examined for histopathology: eyes, proximal portion of the right coronary artery, arch and/or thoracic aorta, nuchal and patellar ligaments, lung, skin, muscle and kidney.

### Histopathology and immunohistochemistry

Five μm thick sections from formalin fixed, paraffin embedded tissues were stained with hematoxylin & eosin, and with alcian blue (pH 2.5 and pH 1.0).

The core proteins of decorin and biglycan were identified with rabbit polyclonal antibodies LF-30 or LF-136 to decorin and LF-106 to biglycan (generous gift from Dr. Larry Fisher, NIDCR, NIH [35]. The antibody to the core protein of human aggrecan was a generous gift from Dr. Peter Roughley [36]. We used an immunohistochemistry protocol used by us previously [37]. Briefly, after deparaffinization and rehydration in descending grades of ethyl alcohol, tissue sections were quenched in 0.3% H_2_O_2 _in methanol and non-specific sites were blocked with normal goat serum (Vector laboratories Inc., USA) for 30 minutes. Slides were incubated at 4°C overnight with primary antibodies (1:500 in PBS) to decorin, aggrecan or biglycan. Next day, slides were treated with biotinylated anti-rabbit IgG (H+L) secondary antibody, made in goat (Vector Laboratories Inc.) at dilution 1:1000 in PBS for one hour. Antigen-antibody complexes were detected using an avidin-biotin complex detection system (Vectastain Elite ABC Kit, Vector Laboratories, Burlingame, CA). Slides were stained with DAB Substrate kit (Vector laboratories), rinsed in water and briefly counter stained with hematoxylin and washed in water.

### Electron microscopy

Samples of midmetacarpal regions of SDFTs from a DLSD-affected (horse no. 8, Table 1) and a control (horse no. 1, Table 3), and upper thoracic aorta from a control horse (no. 1, Table 3) and thoracic aorta of two DSLD-affected horse (case no. 9, 11, Table 1) were fixed at 4°C in 2% glutaraldehyde/2% (para)formaldehyde/0.2% picric acid/0.1 M cacodylate buffer, pH 7.2. The fixed tissue was rinsed at 4°C in 0.1 M cacodylate buffer/300 mM sucrose, pH 7.2. Secondary fixation was performed for 1 hr at 4°C in 1% OsO_4_/0.1 M cacodylate buffer, pH 7.2. After dehydration in solutions of increasing ethanol concentration the fixed aortic tissues were infiltrated with propylene oxide and propylene oxide/Epon 812 mixture, and embedded in Epon 812. Sections were positioned on 400 mesh grid, stained with uranyl acetate and lead citrate, and examined using JEOL 100CXII Transmission Electron Microscope.

The dehydrated fixed tendon tissues were infiltrated with acetone/Spurr resine mixtures (50% Spurr for 1 hr, 50 % Spurr overnight, and 100% Spurr twice for 1 hr. The tendon blocks were polymerized for 1 day at 60°C and were sectioned with glass knives on a Leica Reichert Ultramicrotome. Sections were positioned on 400 mesh grids, stained with uranyl acetate and lead citrate, and examined using JEOL JEM-1210 Transmission Electron Microscope.

### Proteoglycan extraction

Midmetacarpal portions of SDFTs from 2 affected horses (no. 4 and 8, Table 1) and from one control horse (no. 1, Table 3) were dissected, minced, defatted with a chloroform:methanol solution (1:1, 6 ml/1.0 g of dry weight sample), and dried using a Speedvac (SVC 100 H; Thermo Savant, Holbrook, NY). The dry tissue was homogenized and extracted twice with 10 volumes of 4 M guanidine HCl extraction buffer for 24 h on a rotator at 4°C as described by us previously [18]. The combined extracts were dialyzed against deionized water three times for 24 h at 4°C.

### Sepharose CL-2B chromatography

Dialyzed extracts were lyophilized and dissolved in 3 ml of 4 M guanidine HCl. Two ml of dissolved sample (at protein concentrations 4.4 mg/ml for horse no. 8 and 5.75 mg/ml for the control horse) were separated on a molecular sieve Sepharose CL-2B column (1.25 × 110 cm, equilibrated and eluted in 4 M guanidine HCl) at 0.1 ml/min [18]. The Vt or total volume of the column was 146 ml and the Vo or void volume was 52 ml as determined by Dextran blue elution.

### SDS-PAGE

Aliquots (30 μg) of Sepharose CL-2B pools were precipitated for 2 hrs at -80°C with 8 volumes of 100% ethanol. The precipitates was dissolved in 15 μl of 100 mM Tris buffer, adjusted to pH 8.0 with concentrated acetic acid and digested with 20 mU of chondroitinase ABC (*Proteus vulgaris*, Sigma, St. Louis, MO) at 37°C for 24 h. Chondroitinase ABC digested samples and non-digested replicate aliquots were separated on 10% SDS-polyacrylamide gels. The gels were stained with silver nitrate [38].

## Authors' contributions

JH conceived and designed the study, supervised and evaluated all experimental aspects, examined all histological slides and wrote the final version manuscript. BK was involved in post-mortem examination, immunohistochemistry and proteoglycan analysis. AK was involved in post-mortem examination, immunohistochemistry and in writing of the manuscript. JHY participated in proteoglycan analysis. POEM was involved in selection of horses, and in clinical evaluation and diagnosis. All authors read and approved the final manuscript.
